# The Role of the Surface Functionalities in the Electrocatalytic Activity of Cytochrome C on Graphene-Based Materials

**DOI:** 10.3390/nano15100722

**Published:** 2025-05-11

**Authors:** Andrés Felipe Quintero-Jaime, Diego Cazorla-Amorós, Emilia Morallón

**Affiliations:** 1 Departamento de Química Física and Instituto Universitario de Materiales de Alicante (IUMA), University of Alicante, Ap. 99, 03080 Alicante, Spain; morallon@ua.es; 2 Bernal Institute and Department of Chemical Sciences, School of Natural Sciences, University of Limerick (UL), V94 T9PX Limerick, Ireland; 3 Departamento de Química Inorgánica and Instituto Universitario de Materiales de Alicante (IUMA), University of Alicante, Ap. 99, 03080 Alicante, Spain

**Keywords:** functionalization, graphene, cytochrome c, electrocatalysis, peroxide

## Abstract

The development of efficient electron transfer between enzymatic elements and the electrode is considered an important issue in the synthesis and design of bioelectrochemical devices. In this regard, the modification of the surface properties is an effective route to obtain a high-performance electrode using enzymatic elements. As we present here, understanding the role of surface functional groups generated by the electrochemical functionalization of graphene-based materials facilitates the design and optimization of effective electroactive bioelectrodes. In this sense, the surface chemistry directly influences the inherent electrocatalytic activity of cytochrome c (Cyt C) toward the electrochemical reduction of H_2_O_2_. Although the surface oxygen groups provide an immobilization matrix for the Cyt C in the pristine graphene oxide, the electrochemical functionalization with N and P species in one step significantly improves the electrocatalytic activity, since they may facilitate an optimal electrostatic interaction and orientation between the electrode material and the redox heme cofactor in the Cyt C, enhancing the electron transfer process. On the other hand, the lack of surface functional groups in the reduced graphene oxide does not favor the electron transfer with the Cyt C immobilized on the surface being completely inactive. Thus, the incorporation of surface groups using electrochemical functionalization with N and P species provokes a remarkable enhancement of the electrocatalytic activity of cytochrome c, up to four times more than the H_2_O_2_ reduction reaction. This demonstrated the effectiveness of the functionalization process and the impact in the electrochemical performance of Cyt C immobilized in graphene-based electrodes.

## 1. Introduction

The implementation of redox enzymes as efficient electrocatalysts has allowed for the design of new disruptive technologies in new applications and fields, including electrochemical biofuel cells (EBFCs) [1] and artificial photosynthesis [2] for energy harvesting devices, electrochemical biosensors, and active catalysts for the synthesis of industrial compounds, such as the production of enzymatic ammonia [3] or the reduction of CO_2_ [4]. One of the key challenges in the development of high-performance bioelectrochemical devices lies in the establishment of an effective electron transfer (ET) pathway between the electrode and the active site of the redox enzyme [5,6], providing a high turnover number, sensitivity, and outstanding electrocatalytic activity of the enzymatic element [1]. In this sense, the arrangement on top of the electrode surface provides an adequate orientation and bonding to the electrode surface, which is extensively studied in order to promote a direct electrode/enzyme communication [7,8]. Unfortunately, direct ET is considerably limited to a few redox enzymes in which the redox cofactor is either close to the surface or more exposed, such as some pyrroloquinoline (PQQ)-based dehydrogenases [9,10], multicopper oxidases, and heme-based enzymes [11], requiring the use of redox mediator species as electron acceptor species to promote the shuttling of electrons to the electrode, such as Os-polymers [1,12]. However, new approaches focus on more efficient enzymatic elements by engineering the redox cofactor by tuning the redox potential or immobilizing redox-like cofactors on the electrode surface [13,14].

Undoubtedly, the interfacial properties of the electrode surface play an important role in the assembly of the biomolecule in a specific orientation in which the electrons can be shuttled from the redox cofactor to the electrode [15]. Initial approaches that employ self-assembled monolayers (SAMs) on gold electrodes have demonstrated that the terminal motifs of the SAM can favor or hinder the ET process of the redox enzyme [16,17,18], as a result of the electrostatic interactions generated. Xu et al. have demonstrated that the optimal orientation of PQQ-aldehyde dehydrogenase is achieved by a covalent attachment through a histidine-tag (His-tag) and the R-COO^−^ terminal groups, generating a fast ET and a short tunneling distance [19]. Following the same principle, functional groups or dopant agents of conjugated polymers or copolymers facilitate the immobilization, even mediating the ET process with biocatalysts [20,21,22]. For example, a higher concentration of cellobiose dehydrogenase is electrically connected to a screen-printed electrode (SPE) modified with PANI by the backbone groups of the polymer, improving the stability and sensitivity 10 times compared to the unmodified SPE [23]. Furthermore, negatively charged poly-(styrenesulfonate) in PEDOT (PEDOT:PSS) produces binding sites with the positively charged lysine residues in cytochrome c (Cyt C), which facilitates the direct ET mechanism with the electrode surface [24,25]. Another remarkable strategy to promote controlled orientation in multicopper oxidase, such as bilirubin oxidase or laccase, involves the use of surface moieties that mimic the enzyme substrate, such as pyrene [26] and napthaquinones [27,28]. Tahar et al. functionalized multiwalled carbon nanotubes (MWCNTs) with naphtylated-like structures using electrochemical grafting that preferentially orients the active sites of the copper cluster T1 in the laccase, increasing the electrocatalytic activity for the oxygen reduction reaction (ORR) [29]. Following same approach, Gandhi et al. functionalized carboxylic hydroquinone (HQ-COOH) species on MWCNTs’ surfaces, generating an optimal immobilization electrode surface for Cyt C and facilitating the direct electron transfer with Cyt C as well as the redox species of the HQ-COOH, which mediated the H_2_O_2_ detection [30]. By the in situ encapsulation of redox enzymes using an oligomer-like matrix [31] or a Cu/Co-MOF [32], researchers have been able to produce both an immobilization matrix as well as a mediated system to promote direct electron transfer with the superior electrocatalytic performance of the enzymatic element toward glucose oxidation or H_2_O_2_ reduction.

Nowadays, nanometer scale interactions between the electrode and the biocatalyst have been considered a crucial aspect to build up highly efficient ET kinetics in bioelectrodes [33,34]. In this regard, nanostructured carbon-based materials rapidly grow as building blocks in bioelectrode synthesis due to their outstanding electrical conductivity, high electron density, and the presence of different active sites, providing facile electron transfer mobility [35]. Regarding this, the use of carbon nanotubes (CNTs) and graphene-based and other carbon-based materials (i.e., activated carbon, carbon nitride, etc.) has been extensively explored for the design of high-performance electrocatalysts for the oxygen reduction reaction [36], alcohol oxidation [37], and the electrosynthesis of valuable products [38,39,40]. Nonetheless, the use of carbon materials has gained great interest in recent decades for designing noninvasive electrochemical biosensor platforms for the detection of lactate, glucose, and alcohol, as well as EBFCs [41,42,43,44].

In this regard, tailoring the surface properties of carbon materials throughout the surface functionalization (non- and covalently attached) is considered a promising alternative to improve the electrocatalytic activity of the biomolecules and the functionalized carbon material electrodes [45,46,47,48]. Among the different methods of functionalization, electrochemical procedures generate a local and controlled surface functionalization without affecting the bulk properties of the carbon materials by the generation of active species that favor the grafting or deposition of a wide variety of functional species, including nitrogen, oxygen, and phosphorus among others [49,50]. The resulting surface functional groups generate binding sites with the glycosylation shell of the redox enzyme, improving the immobilization process [51,52]. In addition, the functionalities grafted on the surface might present an electroactive behavior that can even work as an effective electron mediator between the redox enzyme and the electrode, as has been observed with CNTs modified with poly-phenothiazine and FAD-dependent glucose dehydrogenase [45]. Interestingly, recent studies have proven that surface functionalities in carbon materials constitute an effective electrical wiring that facilitates the electron transfer kinetics [9,53,54].

Hitherto, typical surface modification procedures of graphene-based electrode materials have involved the formation of surface oxygen groups followed by their substitution with amino-terminated groups in a step-by-step methodology to promote a better ET process during the assembling of the redox enzymatic element. Here, we explore the impact of the different surface chemistry of graphene-based materials using a single-step electrochemical functionalization with 4-aminophenyl phosphonic acid (4-APPA) on the electrocatalytic activity of Cyt C, used as a model biomolecule, towards the electrochemical reduction of H_2_O_2_. By understanding the role of surface chemistry in the electrocatalytic activity of Cyt C, we were able to identify the suitable surface functionalities as well as their impact to improve the activity of Cyt C towards H_2_O_2_. In both cases, the removal of oxygen surface groups by the electrochemical reduction of graphene oxide and the surface functionalization with N and P species considerably improve the electrochemical active surface area of the electrode material and electrocatalytic activity for the H_2_O_2_ of the electrode without enzymes. Interestingly, the immobilization of Cyt C demonstrated that the redox molecule requires the presence of some specific surface functionalities to maintain the activity for the electrochemical reduction of H_2_O_2_ due to an unfavored redox cofactor orientation. In fact, the redox activity of Cyt C and the electrocatalytic performance for the H_2_O_2_ reduction reaction is remarkably enhanced with the presence of N and P species instead of single oxygen species as in GO, suggesting an improved interaction between Cyt C and the nitrogen and phosphorus functional groups on the electrode material. The superior electrocatalytic activity observed in the electrochemical functionalization with N and P species proves the viability of this functionalization process in the synthesis of bioelectrodes.

## 2. Experimental Section

All reagents and methodologies for the electrode preparation and electrochemical surface modification of the graphene-based materials are detailed and described in Section S1 in the Supporting Information.

## 3. Results and Discussion

### 3.1. Electrochemical Reduction of GO Under Neutral Conditions

**Figure 1:**
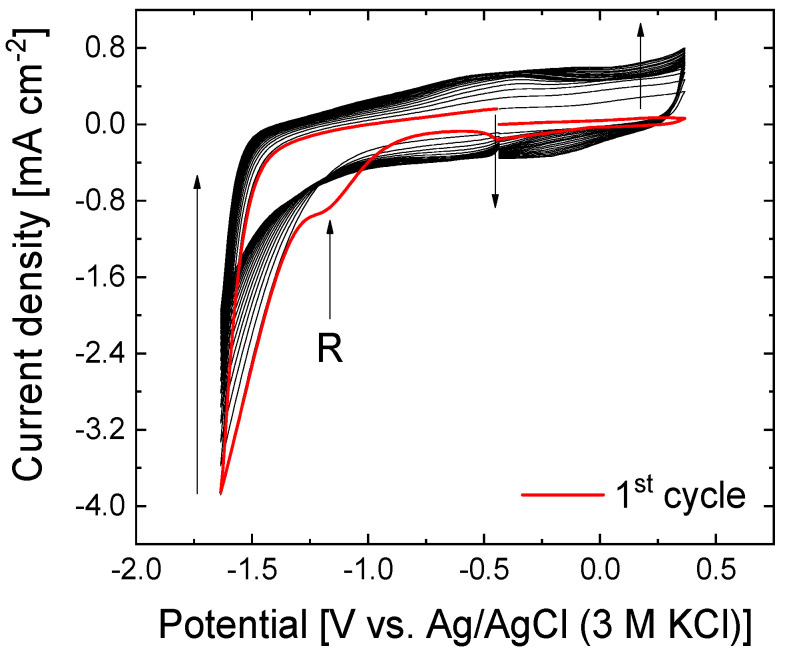
Cyclic voltammograms of the electrochemical reduction of the GO electrode in 0.1 M Na_2_SO_4_ at 50 mV s^−1^ for 20 consecutive cycles. The arrows indicate the evolution with the number of cycles.

Figure 1 shows the voltammogram performed during the electrochemical reduction of GO in an extended potential window (−1.63 to 0.36 V vs. Ag/AgCl (3 M KCl)) at neutral pH conditions. During the first cycle to less positive potentials an irreversible reduction wave (R) at −1.13 V and a high reduction current at the lower potential limit are observed due to the partial reduction of the surface oxygen groups in the GO. The latter behavior is concomitant with a continuous decrease in the current density at lower potentials with potential sweeping. Electrochemical reduction occurs preferentially in the C=O species, decreasing from about 35% to 9% after the electrochemical treatment, as can be observed in the signal of XPS C1s after the electrochemical treatment (see Figure S1 in supporting information) in agreement with Marrani et al. [55]. Furthermore, two different phenomena take place during the electrochemical reduction of GO. The first one involves the increase in the voltammetric charge associated with the formation of the double layer and the second includes the appearance of one redox process at −0.49 V during the positive scan, in which the intensity increases with cycling, which is associated with the redox activity of remaining oxygen functionalities. After 20 cycles under these conditions, the reduced graphene oxide is obtained (rGO).

The electrochemical characterization for both GO and rGO in 0.5 M H_2_SO_4_ between −0.2 and 0.8V (see Figure S1A,C in Supporting Information) shows a tilted voltammogram in GO electrodes, corresponding to a carbon material with low conductivity, as well as a low value of the double layer capacitance. However, the voltammogram of rGO shows important differences with respect to the GO. First, an important increase in the current associated with the double layer is observed, which is related to a higher surface area after the electrochemical reduction. Furthermore, a redox process is clearly observed at around 0.5 V in the positive scan, with a separation of peak potential of 0.4 V, which can be related to the remaining electroactive surface oxygen groups in the graphene layer [56,57].

### 3.2. Electrochemical Surface Functionalization with 4-APPA of GO and rGO

**Figure 2:**
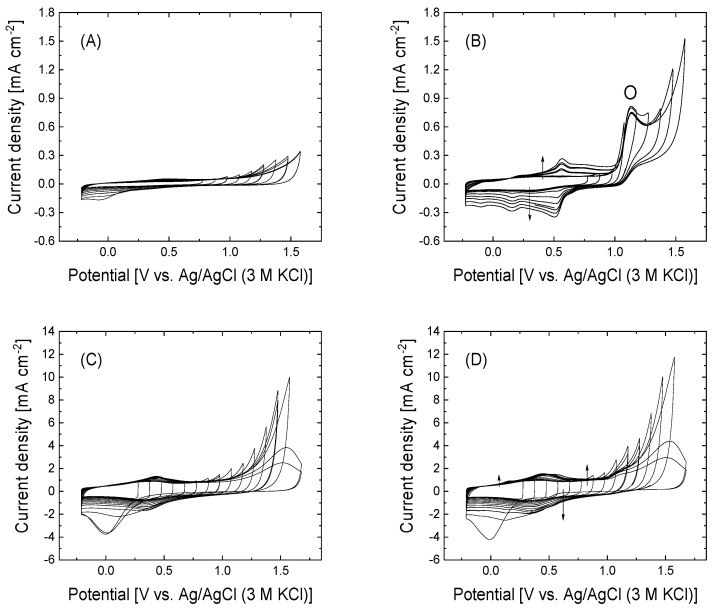
Cyclic voltammograms during the first cycle of the open stepwise potential window for (**A**) GO in 0.5 M H_2_SO_4_, (**B**) GO in 0.5 M H_2_SO_4_+ 1 mM 4-APPA, (**C**) rGO in 0.5 M H_2_SO_4_, and (**D**) rGO in 0.5 M H_2_SO_4_+ 1 mM 4-APPA. 50 mV s^−1^. The arrows indicate the evolution with the number of cycles.

Figure 2 shows the cyclic voltammograms at the upper potential limit stepwise opened from 0.78 to 1.58 V in the absence of 4-APPA (Figure 2A,C) and in the presence of 4-APPA (Figure 2B,D) for GO and rGO, respectively.

In the presence of 4-APPA, GO and rGO present different voltammetric behavior. In the case of GO, the oxidation of 4-APPA shows a clear irreversible oxidation peak at approximately 1.13 V. After this oxidation peak, several reduction processes at 0.53, 0.13, and −0.10 V appear in the voltammograms of GO during the reverse scan associated with the redox activity of the surface functionalities on the surface, with a lower reversibility in comparison with previous results in carbon nanotubes [50]. In contrast, the voltammetric profiles of the rGO in the presence of 4-APPA are very similar to those obtained with rGO in the absence of 4-APPA, suggesting that the contribution of the oxidation of 4-APPA is less important compared to the oxidation of the carbon material. Indeed, rGO does not show significant redox processes compared to those observed during the rGO oxidation in the absence of 4-APPA (see Figure 2C), suggesting the growth and deposition of oligomer layers occur to a lesser extent than in the case of GO, being more important for the oxidation of the rGO than the oxidation of 4-APPA, as has been observed in oxidized carbon nanotubes [58].

Based on the study of the stepwise upper potential limit, two upper potential limits (1.28 and 1.58 V) were chosen for the functionalization of the materials (see Figure S2 in Supporting Information). Once the electrochemical modification with 4-APPA is carried out, a clear oxidation peak is observed in GO; however, this oxidation overlaps with the oxidation of the carbon material in the case of rGO. Interestingly, the clear irreversible oxidation current observed in the case of GO at 1.13 V has not been observed in previous studies with oxidized CNTs [58]. The electrochemical characterization by cyclic voltammetry in 0.5 M H_2_SO_4_ for GO after the electrochemical modification (see Figure S2 in the Supporting Information) shows the appearance of different redox processes at potentials of 0.44, 0.28, and −0.09 V. The increase in the upper potential limit during the 4-APPA oxidation does not produce an important change in the voltammetric profile; only a decrease in the voltammetric charge can be observed, which might be related to the degradation of the oligomers produced during the oxidation of 4-APPA [59,60,61,62]. For rGO, no significant differences can be observed in the voltammograms with respect to those obtained after oxidation in the absence of 4-APPA (see inset plot in Figure S2 in Supporting Information), and only a small increase in the voltammetric charge can be observed when the oxidation is produced in the presence of 4-APPA. In this case, a small and reversible redox process is observed at −0.10 V after the electrochemical treatment in the presence of 4-APPA at both upper potential limits. However, the functionalization of rGO at 1.8 V tends to produce an important oxidation of the carbon material to a greater extent than the oxidation of 4-APPA, generating a tilted voltammogram due to an electrical resistive behavior, as a result of the oxidation of the graphene-like structure.

The XPS analysis for the GO and rGO-modified electrodes shows the amount of N and P species incorporated after the electrochemical functionalization and their chemical nature. Table 1 summarizes the amount of O, N, and P incorporated in graphene-based materials after electrochemical modification.

It can be observed that for GO the amount of nitrogen species increases with the upper potential limit from 1.28 to 1.58 V (Table 1). Nevertheless, a high polarization above 1.28 V causes a decrease in the phosphorus content, due to the overoxidation of the polymeric species formed during the oxidation and the oxidation of the phosphonic groups to phosphoric groups that can be hydrolyzed and released to the electrolyte solution. The XPS data of N and P for rGO show a less important functionalization of the graphene-based material with both species (N and P). Nevertheless, the functionalization of rGO shows an unclear dependence with the upper potential limit, suggesting the saturation of the available sites in the rGO. This behavior is in agreement with the electrochemical response of these electrodes (see Figure S2).

The XPS analysis of both functionalized graphene-based materials demonstrates differences in the chemical nature of the incorporated functional groups. The XPS N1s spectra for GO-4APPA (see Figure S3 in the Supporting Information) can be deconvoluted into two peaks with a binding energy of around 400 and 402 eV. The fist peak at 400 eV can be assigned to the presence of amine-like groups, as a consequence of the formation of oligomer/polymeric chains [63,64,65,66]. The second contribution at 402 eV increases with the increasing of the upper potential limit due to oxidation of N species into oxidized nitrogen species, specifically positively charged nitrogen species (N^+^) due to protonated nitrogen functionalities (-NH_2_^+^/-NH^+^=) during the oxidative electrochemical functionalization [61,63]. On the other hand, the XPS N1s spectrum of rGO-4APPA shows a small additional contribution at a lower energy binding at around ~398.5 eV related to the presence of imines as well as a non-variation in the distribution of the nitrogen species after the functionalization (see Table S1 in Supporting Information).

**Figure 3:**
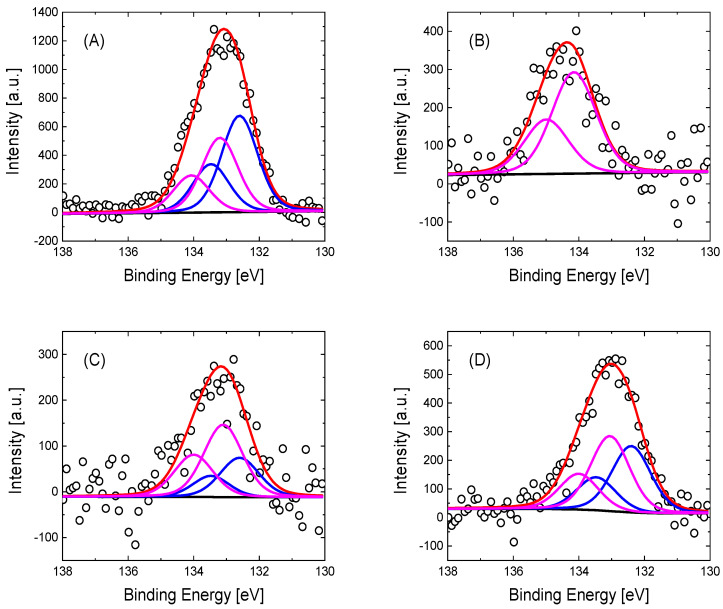
P2p XPS spectra for GO and rGO electrochemically modified with 4-APPA at different oxidation potentials: (**A**) GO-4APPA modified at 1.28 V, (**B**) GO-4APPA modified at 1.58 V, (**C**) rGO-4APPA modified at 1.28 V, and (**D**) rGO-4APPA modified at 1.58 V.

Regarding the XPS P2p spectra, the deconvolution of the phosphorus species can be performed in one or two doublets, as can be observed in Figure 3. For GO-4APPA and rGO-4APPA electrodes modified at a potential of 1.28 V, the spectra show a first contribution at 132.6 eV, which is associated in the literature with the binding energy of C-P species [67,68], in agreement with the presence of the phosphonic group of 4-APPA. Furthermore, a second contribution at 133.3 eV, related to the P-O species, increases with the upper potential limit, as a consequence of the oxidation of the phosphonic group to phosphate or phosphoric groups: (-O-P(OH)_2_). However, when GO is electrochemically modified at a potential of 1.58 V, the phosphorus species shows an important shift to a higher binding energy at 134.1 eV, which is related to the oxidized phosphorus species (see Table S1 in supporting information) in which a greater oxygen interaction is present, in agreement with values reported in the literature for this species in carbon materials [63,66,69]. Interestingly, the presence of the P species on the electrode surface might suggest a negatively charged surface of the graphene-based material after the functionalization process.

The electrochemical and physicochemical characterization of pristine and electrochemically functionalized graphene-based materials have demonstrated that the functionalization with N and P species at 1.58 V tends to produce a partial desorption of the surface functionalities, especially the phosphorus groups. Additionally, the degradation process of the electrode surface by the electrooxidation generates important damage to the pristine GO and rGO structures and their properties. Thus, in the following sections, pristine and functionalized graphene-based materials at 1.28V have been evaluated as electrodes for the immobilization of cytochrome C and for the evaluation of their electrochemical activity.

### 3.3. Electrochemical Behavior at Neutral pH

**Figure 4:**
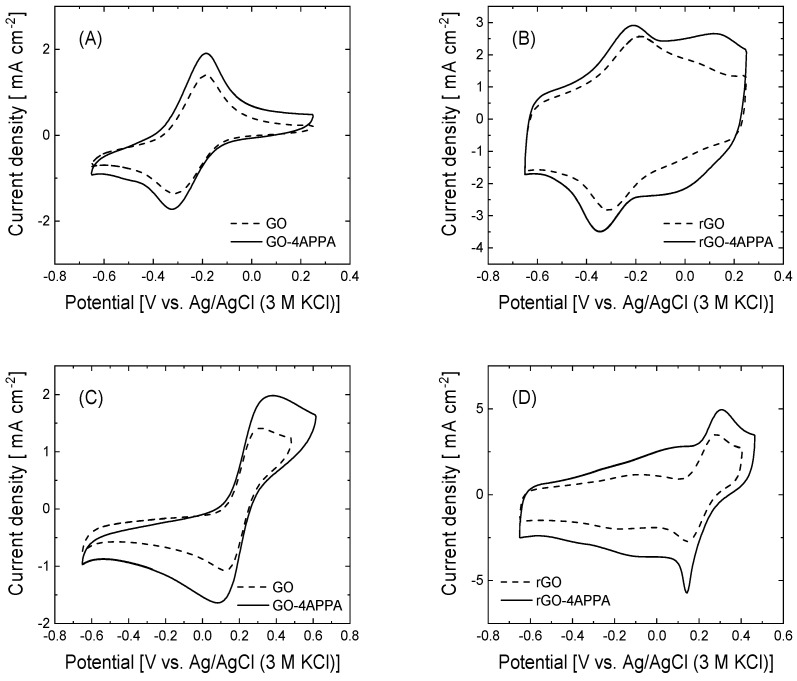
Cyclic voltammograms at 50 mV s^−1^ of graphene-based materials electrochemically modified with 4-APPA in 0.1 M PBS (pH = 7.2): (**A**,**B**) 1 mM [Ru(NH_3_)_6_]^3/4+^ and (**C**,**D**) 5 mM ([Fe(CN)_6_]^3/4−^).

The properties of the electrode/electrolyte interface are strongly influenced by the chemical nature of the electrode surface, which can favorably affect the electrochemical activity of the electrode material. In this context, the influence of the electrode surface on pristine and electrochemically modified graphene-based materials has been evaluated by cyclic voltammetry in the presence of a positive ([Ru(NH_3_)_6_]^3/4+^) and negative ([Fe(CN)_6_]^3/4−^) redox probe. Figure 4 shows the cyclic voltammograms of GO-4APPA and rGO-APPA (solid line) and the pristine graphene-based materials (dotted line) in the presence of each redox probe.

In all the cyclic voltammograms of graphene-based materials, the quasi-reversible redox couple corresponding to the one electron transfer processes of [Ru(NH_3_)_6_]^3/4+^ and [Fe(CN)_6_]^3/4−^ are clearly observed in the cyclic voltammograms. The analysis of both the cathodic and anodic peak currents of the processes shows a linear dependence with the square root of the scan rate (see Figures S4 and S5 in Supporting Information), corresponding with a diffusion-controlled process. The linear fitting of the I_peak_ vs. *v*_scan_^1/2^ can be expressed as a function of the active surface area of the electrode available for the electron transfer of the redox species following the Randles–Sevcik equation [70] (Equation (1)):

*I_p_ =* 2.69 × 10^−5^
*· n*^3/2^ · *A · D*_0_^1/2^ · *C*_0_* · v_scan_^1/2^ (1)

where *Ip* (A) is the peak current of the process; *n* is the number of exchanged electrons; *A* is the electroactive surface area (cm^2^); *D*_0_ is the diffusion coefficient of the electroactive species (cm^2^ s^−1^), which has been reported to be about 6.70 × 10^−6^ and 7.65 × 10^−6^ cm s^−1^ for [Ru(NH_3_)_6_]^3/4+^ and [Fe(CN)_6_]^3/4−^ [71,72], respectively; *C*_0_^*^ is the concentration of the electroactive species (mol cm^−3^); and *v_scan_* is the scan rate (V s^−1^). The estimation of the electroactive surface area employing the Randles–Sevcik relation, as can be observed in Table 2, shows an important ca. 3.8 times increase once the electrochemical reduction of GO takes place. In addition, an increase in the active surface area is also observed in both graphene-based materials after the electrochemical functionalization with the 4-APPA, reaching up to 37 and 60% of the pristine active surface area for the [Ru(NH_3_)_6_]^3/4+^ and [Fe(CN)_6_]^3/4−^, respectively, which is in agreement with the increase in the double layer charge in the cyclic voltammograms after the electrochemical functionalization (see Figure S2). These results can be interpreted taking into account that the mechanism of the functionalization process includes the formation of both covalently attached molecules of 4-APPA and non-covalently adsorbed oligomer chains of 4-APPA. Then, the deposited functional groups and oligomer structures can provide a greater number of active sites in which the electron transfer process might take place at higher rates.

The [Ru(NH_3_)_6_]^3/4+^ and [Fe(CN)_6_]^3/4−^ are two typical outer-sphere redox probes vastly employed in the study of the electrocatalytic activity of electrode materials, which have a high dependence on the nature of the electrode material. Therefore, the kinetics of the electron transfer process can be influenced by the chemical surface of the electrode material. From the voltammogram profiles in Figure 4, in the peak separation (∆E) of both the redox probe and the values of the heterogeneous rate constant (*k*_0_), determined following the Nicholson and Mahe methods [73,74], different electrochemical behaviors, depending on the redox probe and the graphene-based material, can be observed (Table 2).

The peak separation of the [Ru(NH_3_)_6_]^3/4+^ redox couple presents a negligible variation of about 10 mV for all the electrode materials evaluated, suggesting that the electron transfer process is not significantly affected by the incorporation of the 4-APPA functionalities. In contrast, the peak separation values for the [Fe(CN)_6_]^3/4−^ increase to about 80 and 17 mV once the GO and rGO are functionalized with the 4-APPA. Taking into account the different functional groups generated, specifically the N and P species, a large part of the nitrogen groups in the functional species are in their neutral state at neutral pH conditions [75]. On the other hand, the phosphonic and phosphate groups are fully deprotonated, increasing the net negative charge of the surface of the graphene-based materials. Then, electrostatic repulsion between the [Fe(CN)_6_]^3/4−^ and the modified electrode surface is more important, affecting the concentration of the redox species near the electrode surface. This behavior is more notable on the GO-4APPA electrodes, where the highest amounts of phosphorus groups are incorporated on the surface, causing an important increase in the potential peak separation (Table 2). The values of k_0_ are shown in Table 2. It can be observed that the constant rate values for [Fe(CN)_6_]^3/4−^ decrease in the functionalized graphene-based materials with 4-APPA. For instance, the value of *k*_0_ in GO-4APPA decreases more by than four times with respect to GO. In the case of [Ru(NH_3_)_6_]^3/4+^, the effect of the functionalization is lower than the values in [Fe(CN)_6_]^3/4−^. Therefore, the insertion of different functionalities that modify the net surface charge can promote the immobilization of biocatalytic elements, specifically positively charged enzymes, as well as the effective electrochemical activity of the biocatalyst.

### 3.4. Electrocatalytic Activity of Cyt C Immobilized on Functionalized GO and rGO

**Figure 5:**
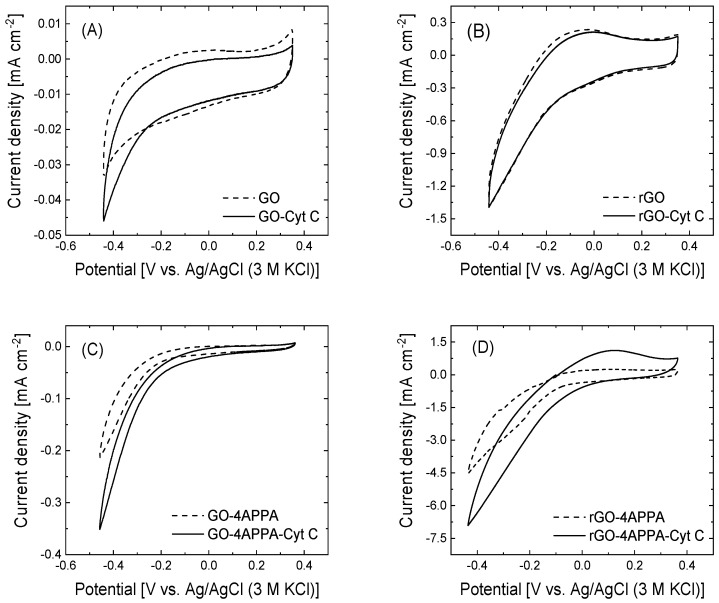
Cyclic voltammograms in 0.1 M PBS (pH = 7.2) + 100 mM H_2_O_2_ for (**A**) GO, (**B**) rGO, (**C**) GO-4-APPA, and (**D**) rGO-4-APPA before (dash line) and after (solid line) immobilization of Cyt C at 20 mV s^−1^ under room atmosphere.

The electrocatalytic activity of the Cyt C immobilized on the graphene-based electrodes has been evaluated regarding the electrochemical reduction of H_2_O_2_ (Figure 5). For the analysis of the electrocatalytic activity, the electrochemical characterization of the different electrodes in absence of H_2_O_2_ has been performed (see Figure S6 in supporting information), showing modifications in the double layer contribution after the immobilization of Cyt C. On the other hand, the pseudocapacitive contribution of the surface functionalities (oxygen, nitrogen, and phosphorus species) tends to produce a hindrance of the redox process associated with the heme redox cofactor; thus, the electrocatalytic activity toward H_2_O_2_ has been used to evaluate the electrocatalytic activity.

The cyclic voltammograms of the pristine GO and rGO show that both electrode materials have a catalytic activity toward the electrochemical reduction of H_2_O_2_, which reaches variations in the current density of 6.5 × 10^−3^ mA cm^−2^ and 1.14 mA cm^−2^ for GO and rGO at −0.43 V, respectively. A notable improvement in the electrocatalytic activity towards the H_2_O_2_ reduction for rGO can be understood considering the higher electrochemical active surface area (Table 2) [55,76,77]. Once both materials are electrochemically functionalized with 4-APPA, the electrocatalytic activity toward the H_2_O_2_ reduction is dramatically improved, reaching a current density variation value of about 0.18 mA cm^−2^ and 4.2 mA cm^−2^ for GO and rGO, respectively, which is approximately six and three times the current density variation in the pristine electrodes. According to our previous observation (see Table 2), the incorporation of nitrogen and phosphorus surface functionalities provides an increase in the electroactive surface area as well as the presence of active sites for the electrocatalytic reaction of hydrogen peroxide.

Depending on the electrode employed as the support for the immobilization of the Cyt C, the electrocatalytic activity is enhanced or almost unchanged following the reaction pathway indicated in Equations (2) and (3). In the case of the GO-Cyt C electrode, the variation in the current density in the presence of hydrogen peroxide starts at an onset potential (E_onset_) of about −0.2 V, which is 100 mV more positive than electrodes without the biomolecule. The maximum current value was 16.5 × 10^−3^ mA cm^−2^ at −0.43 V, which is up to 2.5 times higher than the catalytic activity of the pristine GO without the immobilized biomolecule. Then, Cyt C maintains its catalytic activity on this electrode surface after the immobilization process. Interestingly, after the immobilization of Cyt C in 4-APPA functionalized GO electrodes, the variation in the current density related to the H_2_O_2_ electroreduction has an additional contribution of 0.15 mA cm^−2^ to the current density values, compared to the same electrode without Cyt C, suggesting an improvement in the catalytic activity of around 83%. This increase in the current is directly associated with the current generated by the biomolecule on the electrode. In comparison, the current density contribution associated with Cyt C in the unmodified GO (about 0.01 mA cm^−2^) is 15 times lower than the activity of Cyt C in the GO-4APPA electrode. In this sense, the improvement in the electrocatalytic activity of Cyt C in the functionalized GO is mainly caused by the interactions between the surface functional groups and the amino acid residues in the structure of Cyt C which produces its adequate orientation towards the electrode.

Chemical pathway: ½ H_2_O_2_ + H^+^ + Cyt C [Heme Fe(II)]→ H_2_O + Cyt C [Heme Fe(III)] (2)

Electrochemical pathway: Cyt C [Heme Fe(III)] + 1e^−^→ Cyt C [Heme Fe(II)] (3)

Surprisingly, rGO and rGO-Cyt C show the same current density variation in the presence of H_2_O_2_ in the solution, which indicates that the Cyt C does not contribute to the electrochemical reduction of H_2_O_2_. This behavior might indicate that the interaction between immobilized Cyt C and the surface of rGO generates a biomolecule assemblage in which the electrical communication between the redox cofactor of Cyt C and the electrode is not favored. The incorporation of the surface functionalities of the N and P species in rGO provokes a dramatic improvement in the electrocatalytic activity in the presence of H_2_O_2_ due to a larger electrochemical surface area, as has been observed for GO. Following the immobilization of Cyt C in the rGO-4APPA electrodes (see Figure 5D), the cyclic voltammogram in the presence of the H_2_O_2_ displays two important features: a positive shifting at around 100 mV in the onset potential for the electrochemical reduction of the H_2_O_2_ as well as an increase of 58% in the current density variation in the presence of H_2_O_2_. The latter can be associated with the current generated due to the immobilized Cyt C. The current contribution of the Cyt C in the functionalized rGO electrode material demonstrates an effective improvement in the electrical communication between the electrode and the biomolecule through the surface functional groups. Previous works have demonstrated that hydrophilic and negatively charged groups can interact directly with the lysine residues, which might favor the electron transfer with the heme cofactor in the Cyt C [25,78]. In this sense, the deprotonated phosphonic/phosphoric groups under the pH conditions of the immobilization of Cyt C (isoelectric point ≈ 9.5) [79] produces a net negative charge of the electrode surface, which results in a strong electrostatic interaction with the positively charged Cyt C. In this sense, the arrangement of the biomolecule redox cofactor can be close to the electrode surface, as has been observed with other redox biomolecules [9,15].

**Figure 6:**
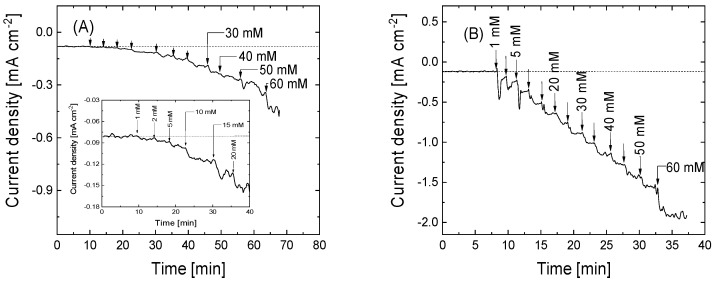

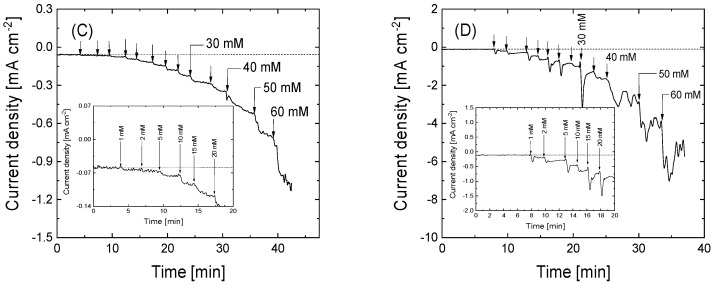
Chronoamperometric profiles under stirring conditions at −0.4 V vs. Ag/AgCl (3 M KCl) in room atmosphere for graphene-based electrode materials electrochemically functionalized with 4-APPA with and without immobilized Cyt C: (**A**) GO-4APPA, (**B**) rGO-4APPA, (**C**) GO-4APPA-Cyt C, and (**D**) rGO-4APPA-Cyt C. Inset: enlargement of low concentration region.

**Figure 7:**
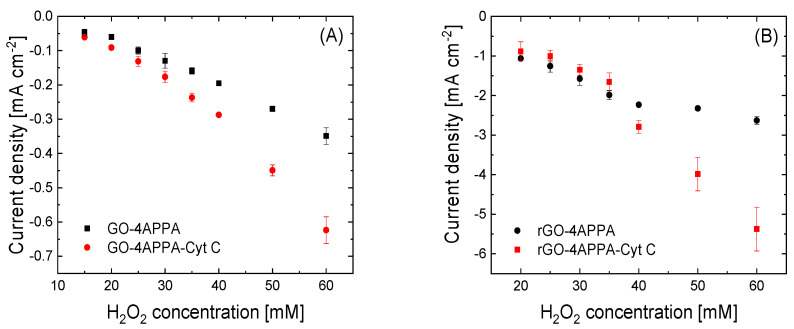
Calibration curves toward H_2_O_2_ reduction obtained for all graphene-based electrode materials electrochemically functionalized with 4-APPA for (**A**) graphene oxide electrodes and (**B**) electrochemically reduced graphene oxide electrodes. Measurements were carried out in triplicate.

Figure 6 displays the chronoamperometric profiles of the different functionalized electrode materials during the continuous addition of H_2_O_2_ aliquots. As can be observed in all electrode materials, the addition of H_2_O_2_ produces a rapid increase in the negative current only 10 s after the addition, which is associated with the electrochemical reduction of H_2_O_2_. These changes are more important for the electrodes in which Cyt C has been immobilized. In this sense, the increase in the current density values shows an enhancement in the electrocatalytic activity of the electrodes GO-4APPA and rGO-4APPA of two and five times the maximum current density value at 60 mM H_2_O_2_, respectively. Figure 7 shows the calibration curves for the different electrodes. Table 3 contains the most relevant parameters for the detection of H_2_O_2_, in which the sensitivity, LOD, and LOQ are presented. Furthermore, a comparison of the electrochemical parameters with similar bioelectrodes, as reported in the literature, based on Cyt C, is presented in Table S2 in the Supporting Information.

As expected, the sensitivity and reduction current density of H_2_O_2_ increase significantly with the electrochemical reduction of GO in rGO and with its further electrochemical functionalization with 4-APPA (see Figure 7B). In this regard, the decrease in the surface oxygen groups after the electrochemical reduction and the incorporation of negatively charged functionalities (phosphonic/phosphoric groups) reduce the hindrance effect, promoting a better electrical communication with the heme cofactor in the Cyt C, as well as the electron mobility over the electrode due to the decrease in the concentration of defects in the graphene layer of the rGO.

## 4. Conclusions

The presence and nature of surface functional groups in graphene-based electrode materials have demonstrated an important influence in the immobilization and redox activity of Cyt C, promoting an optimal interaction of the biomolecule and promoting a more efficient electrical communication between the electrode surface and the heme redox cofactor. In the case of graphene oxide, the incorporation of negatively charged phosphonic/phosphoric groups at a neutral pH facilitates the interaction with positively charged residues in the Cyt C during the immobilization process, maintaining the activity of the biomolecule. In this sense, the good compatibility of the functionalized graphene-based materials provides a suitable platform for the immobilization of the Cyt C. At the same time, this interaction produces an enhancement in the electrocatalytic activity toward the H_2_O_2_ reduction, which is observed as an increase in the catalytic current density associated with the electrochemical reduction of H_2_O_2_. Furthermore, the partial elimination of surface oxygen groups in pristine GO electrochemically induces an enhancement in the electroactive surface area and the electrocatalytic activity of the electrode. Nevertheless, a low concentration of functional groups hinders the redox activity of the Cyt C, avoiding the electron transfer between the rGO and the Cyt C. The functionalization of the rGO electrode with 4-APPA modulates the surface chemistry and improves the immobilization and electrical communication of the biomolecule with the electrode, reaching current density values of up to 5.5 mA cm^−2^, which are 10 times higher than the GO modified with 4-APPA. Therefore, depending on the surface chemistry in graphene-based materials the catalytic performance of bioelectrodes can promote an effective interconversion of the redox element, a higher catalytic current density, and low overpotentials, which are ideal for the fabrication of bioelectrodes as was observed with the modified graphene-based electrode with P and N species. In this regard, the electrochemical functionalization process with 4-APPA represents an alternative to the conventional processes of multi-step bioelectrode fabrication, due to the remarkable compatibility with the biomolecule elements and the great performance achieved.

## Figures and Tables

**Table 1:** Chemical composition obtained from XPS of electrochemically modified graphene-based materials with 4-APPA.

Graphene-Based Material	Upper Potential Limit [V vs. RHE]	O (at%)	N (at%)	P (at%)	C (at%)
GO	Pristine	34.8	0.28	--	64.92
	1.28	22.2	0.71	1.14	75.95
	1.58	29.7	1.54	0.61	68.15
rGO	Pristine	18.7	0.39	--	80.91
	1.28	23.0	1.37	0.52	75.11
	1.58	21.5	1.03	0.65	76.82

**Table 2:** Electrochemical parameters of peak separation (∆E), formal potential (E^0^), electroactive surface area (A), and heterogeneous rate constant (*k*_0_) for [Ru(NH_3_)_6_]^3/4+^ and 5 mM [Fe(CN)_6_]^3/4−^ redox probe performed with electrochemically modified graphene-based materials with 4-APPA.

Graphene-Based Electrode Material	$A_{{Ru}^{2+/3+}}$ **[cm^2^]**	$A_{{Fe}^{2+/3+}}$ **[cm^2^]**	$∆E_{{Ru}^{2+/3+}}$ [mV] *	$∆E_{{Fe}^{2+/3+}}$ [mV] *	$E_{{Ru}^{2+/3+}}^{0}$ [V] *	$E_{{Fe}^{2+/3+}}^{0}$ [V] *	$k_{0}^{{Ru}^{2+/3+}}$ **[×10^−3^ cm s^−1^]**	$k_{0}^{{Fe}^{2+/3+}}$ **[×10^−3^ cm s^−1^]**
GO	0.444	0.045	126	150	−0.250	0.216	2.70	1.38
GO-4APPA	0.609	0.075	136	230	−0.254	0.253	1.82	0.28
rGO	1.680	0.228	129	111	−0.247	0.212	1.16	2.12
rGO-4APPA	2.003	0.399	137	128	−0.279	0.221	0.98	1.34

* Parameters have been determined at 100 mV s^−1^.

**Table 3:** The figures of merit for the electrocatalytic activity of the graphene-based electrode material functionalized with 4APPA and Cyt C.

Graphene-Based Electrode Material	LOD [mM] *	LOQ [mM] **	Sensitivity [μA mM^−1^ cm^−2^]
GO-4APPA	0.1	0.33	6.65
GO-4APPACyt C			9.20
rGO-4APPA			44.30
rGO-4APPA-Cyt C			111.68

* The LOD was determined experimentally as the H_2_O_2_ concentration that produces a notable current variation in the background current. For comparative purposes, 1 mM was selected as the initial concentration. ** LOQ = 3.3LOD.

## Data Availability

The raw data supporting the conclusions of this article will be made available by the authors on request.

## Supplementary Materials

The following supporting information can be downloaded at: https://www.mdpi.com/article/10.3390/nano15100722/s1. Figure S1. (A) Cyclic voltammogram for GO in 0.5 M H_2_SO_4_ at 50 mV s^−1^ under N_2_ atmosphere. (B) XPS spectra of C1s signal for GO. (C) Cyclic voltammogram for rGO in 0.5 M H_2_SO_4_ at 50 mV s^−1^ under N_2_ atmosphere. (D) XPS spectra of C1s signal for rGO after the electrochemical reduction; Figure S2. Cyclic voltammograms obtained during 10 cycles for a GO and rGO electrodes in 0.5 M H_2_SO_4_ (solid lines) and 0.5 M H_2_SO_4_ + 1 mM 4-APPA (dash lines) at 10 mV s^−1^ under N_2_ atmosphere at different positive potential limits: (A) GO-1.28 V, (B) GO-1.58 V, (C) rGO-1.28 V, and (D) rGO-1.58 V. Inset: Steady state CVs in 0.5 M H_2_SO_4_, for graphene-based materials electrochemically modified in absence (dash line) and in presence (solid line) at v_scan_ = 50 mV s^−1^; Figure S3. N1s XPS spectra deconvoluted for GO and rGO electrochemical modified with 4-APPA at different oxidation potential: (A) GO modified at 1.28 V, (B) GO modified at 1.58 V, (C) rGO modified at 1.28 V and (D) rGO modified at 1.58 V; Table S1. Chemical distribution of the N and P species obtained from XPS of electrochemical modified graphene-based materials with 4-APPA; Figure S4. Cyclic voltammograms at different scan rates (10, 20, 50, 100, 200, and 500 mV s^−1^) of the pristine graphene-based materials in 0.1 M PBS (pH = 7.2): (A,B) 1 mM [Ru(NH_3_)_6_]^3/4+^ and (C,D) 5 mM ([Fe(CN)_6_]_3/4_^−^); Figure S5. Cyclic voltammograms at different scan rates (10, 20, 50, 100, 200, and 500 mV s^−1^) of the graphene-based materials electrochemically modified with 4-APPA in 0.1 M PBS (pH = 7.2): (A,B) 1 mM [Ru(NH_3_)_6_]^3/4+^ and (C,D) 5 mM ([Fe(CN)_6_]^3/4−^); Figure S6. Cyclic voltammograms of electrochemical modified GO and rGO before and after the immobilization of Cyt C in 0.1 M PBS (pH = 7.2) at 20 mV s^−1^; Table S2. Comparative of the electrochemical parameters of bioelectrodes reported in literature. See Refs. [28,30,50,80,81,82,83,84,85,86,87].
