# Supplementary material for: The Role of the Surface Functionalities in the Electrocatalytic Activity of Cytochrome C on Graphene-Based Materials
Source: Nanomaterials (Basel). 2025 May 11;15(10):722. doi: 10.3390/nano15100722 (PMC12113858; doi:10.3390/nano15100722)
Supplement: Supplementary file 1 [file nanomaterials-15-00722-s001.zip › nanomaterials-3584394-supplementary.pdf]

Supporting Information

THE ROLE OF THE SURFACE FUNCTIONALITIES IN THE ELECTROCATALYTIC ACTIVTY OF CYTOCHROME C ON GRAPHENE-BASED MATERIALS.

Andrés Felipe Quintero-Jaime[a,c,*], Diego Cazorla-Amorós[b,*], Emilia Morallón[a].

[a] Departamento de Química Física and Instituto Universitario de Materiales de Alicante (IUMA), University of Alicante, Ap. 99, 03080, Alicante, Spain

[b] Departamento de Química inorgánica and Instituto Universitario de Materiales de Alicante (IUMA), University of Alicante, Ap. 99, 03080, Alicante, Spain

[c] Bernal Institute and Department of Chemical Sciences, School of Natural Sciences, University of Limerick (UL), Limerick V94 T9PX, Ireland.

[*] Corresponding author: andres.quintero@UL.ie / cazorla@ua.es

S1. Experimental

1.1. Reagents

Sulphuric acid (98%) analytical reagent was obtained from VWR Chemicals. 4-amino phenyl phosphonic acid (4-APPA, +98%), used as modifier agent, was purchased from Tokyo Chemical Industry co (TCI). Sodium sulfate (Na_2SO_4) (99%) was obtained from Merck. Potassium dihydrogen phosphate (KH_2PO_4) and dipotassium hydrogen phosphate tri hydrate ($K_2HPO_4{\cdot}3H_2O$), obtained from VWR Chemicals, were used to prepare phosphate buffer solution. Cytochrome C from bovine heart (Cyt C, +95%, 12.327 kDa) and hydrogen peroxide (30% w/w in H_2O_2) have been obtained from Sigma Aldrich. All the solutions were prepared using ultrapure water (18 MOhms cm, Purelab Ultra Elga equipment). The N_2 (99.999%) was provided by Air Liquide.

1.2. Synthesis of graphene-based materials

1.2.1. Graphene oxide

Synthesis of graphene oxide (GO) was performed using the modified Hummers method [80]. Graphite powder is treated under acidic and oxidizing conditions to promote the exfoliation of the graphene-sheets, incorporating high amount of surface oxygen groups, making easy the dispersion in polar media.

The working electrode for electrochemical modification was prepared using glassy carbon (GC), as support, modified with the GO. 1 mg of GO was dispersed in ultrapure water employing an ultrasonic cold-bath for 45 minutes, achieving a dispersion of 1 mg mL^{-1} of GO. Prior to the casting, the glassy carbon electrode surface (3 mm diameter) was sanded with emery paper and polished using 1 and 0.05 µm alumina slurries, then rinsed with ultrapure water. Afterwards, 5 µL aliquot of the dispersion was dropped onto the glassy carbon surface and dried under an infrared lamp to remove the solvent. This procedure was repeated twice.

1.2.2. Electrochemical reduction of Graphene oxide

The electrochemical reduction of GO was carried out by cyclic voltammetry conditions using an eDAQ Potentiostat (EA163 model) coupled to a EG&G Parc Model 175 wave generator and the data acquisition was performed with a eDAQ e-corder 410 unit (Chart and Scope Software), using a standard three-electrode cell configuration. The glassy carbon electrode modified with GO was the working electrode (WE), a graphite rod as counter electrode (CE) and a Ag/AgCl (3 M KCl) introduced in the working electrolyte, the reference electrode. The procedure consisted in the application of 20 cycles in a potential range between -1.63 to 0.36 V, at a scan rate of 50 mV s^{-1} in a deoxygenated solution of 0.1 M Na_2SO_4 [81]. Afterwards electrodes are washed with excess of ultrapure water and stored for further modification or characterization.

1.3. Electrochemical modification with 4-aminophenyl phosphonic acid

Electrochemical modification of graphene-based materials was performed using an Autolab PGSTAT 302 (Metrohm Netherlands) potentiostat, with a standard three-electrode cell configuration, in which the glassy carbon electrode modified with graphene-based materials was the working electrode (WE), a graphite rod was used as counter electrode and the Ag/AgCl (3 M KCl) introduced in the same electrolyte but without 4-APPA, was used as reference electrode. Electrochemical modification was carried out as previously reported [50].

1.4. Immobilization of Cyt C on modified graphene-based electrodes

For immobilization of Cyt C, 7 µL of 1 mg mL^{-1} of the enzyme previously dissolved in phosphate buffer solution (PBS) were drop-casted onto the electrode surface of the GO-4APPA and rGO-4PPA. Incubation of the electrode was performed at 4ºC during 4 hours. Afterward, electrode modified with Cyt C was washed with excess of 0.1 M PBS in order to remove non immobilized enzyme. Modified electrodes with Cyt C are named as follows: GO-4APPA-Cyt C and rGO-4APPA-Cyt C.

1.5. Electrochemical characterization

Electrochemical characterization of the graphene-based materials unmodified and modified with 4-APPA was evaluated by cyclic voltammetry in acid medium (0.5 M H_2SO_4) and neutral conditions (0.1

M PBS (pH=7.2)), employing a three electrode configuration cell, where, glassy carbon modified with graphene-based materials functionalized with 4-APPA, was the working electrode (WE), a graphite rod was the counter electrode (CE) and a Ag/AgCl (3 M KCl) electrode introduced in the same electrolyte, was the reference electrode (RE). Potential range was fixed between -0.22 and 0.78 V at 50 mV s^{-1}, for all the characterization. Furthermore, graphene-based materials functionalized with 4-APPA were evaluated in neutral conditions (0.1 M PBS (pH=7.2)) with two different redox probe: 1 mM $[Ru(NH_3)_6]^{3/4+}$ and 5 mM $[Fe(CN)_6]^{3/4-}$.

Electrocatalytic activity of immobilized Cyt C in the graphene-based materials functionalized with 4-APPA as well as unmodified electrodes, towards H_2O_2 reduction was evaluated employing in a 3-electrode cell configuration were a graphite rod was the counter electrode (CE), an Ag/AgCl (3 M KCl) (E^0 = 0219 V vs SHE) as reference electrode, and the glassy carbon electrode modified with the corresponding graphene-based material as working electrode. In all cases, meniscus configuration was employed in order to control the active area of the electrode exposed. For electrocatalytic measurements, chronoamperometry was used applying a constant potential at -0.40 V vs. Ag/AgCl (3 M KCl), where the reduction of H_2O_2 takes place. During all measurements stirring conditions and atmospheric conditions are maintained to avoid diffusional limitations and interference of the oxygen reduction reaction, respectively. After stabilization of the current at -0.4 V, different concentrations of H_2O_2 from a stock solution of H_2O_2 are spiked in the electrochemical cell producing a current variation. Time between aliquots was fixed in 5 minutes to ensure a stabilization of the current signal produces by the H_2O_2 reduction reaction.

1.6. Physicochemical characterization

X-Ray photoelectron spectroscopy (XPS) was performed in a VG-Microtech Mutilab 3000 spectrometer using an Al Kα radiation (1253.6 eV). The deconvolution of the XPS peaks for C1s, O2p, P2p and N1s was done by least squares fitting using Gaussian-Lorentzian curves, while a Shirley line was used for the background determination. The P2p spectra have been analyzed considering the spin-

orbit splitting into P2p3/2 and P2p1/2 with a 2:1 peak area ratio and an energy separation of 0.87 eV [82].

S2. Reference

80. Marcano, D.C.; Kosynkin, D. V.; Berlin, J. M.; Sinitskii, A.; Sun, Z.; Slesarev, A.; Alemany, L. B.; Lu, W.; Tour, J. M. Improved Synthesis of Graphene Oxide. *ACS Nano* **2010**, *4*, 4806–4814. https://doi.org/10.1021/nn1006368.

81. Shao, Y.; Wang, J.; Engelhard, M.; Wang, C.; Lin, Y. Facile and controllable electrochemical reduction of graphene oxide and its applications. *J. Mater. Chem.* **2010**, *20*, 743–748. https://doi.org/10.1039/B917975E.

50. Quintero-Jaime, A.F.; Cazorla-Amorós, D.; Morallón, E. Electrochemical functionalization of single wall carbon nanotubes with phosphorus and nitrogen species. *Electrochim. Acta* **2020**, *340*, 135935. https://doi.org/10.1016/j.electacta.2020.135935.

82. Thermo scientific XPS simplified [Online], Thermo scientific XPS, Phosphorus (2020). Available at: https://xpssimplified.com/elements/phosphorus.php#appnotes [Accessed 2018 March 3].

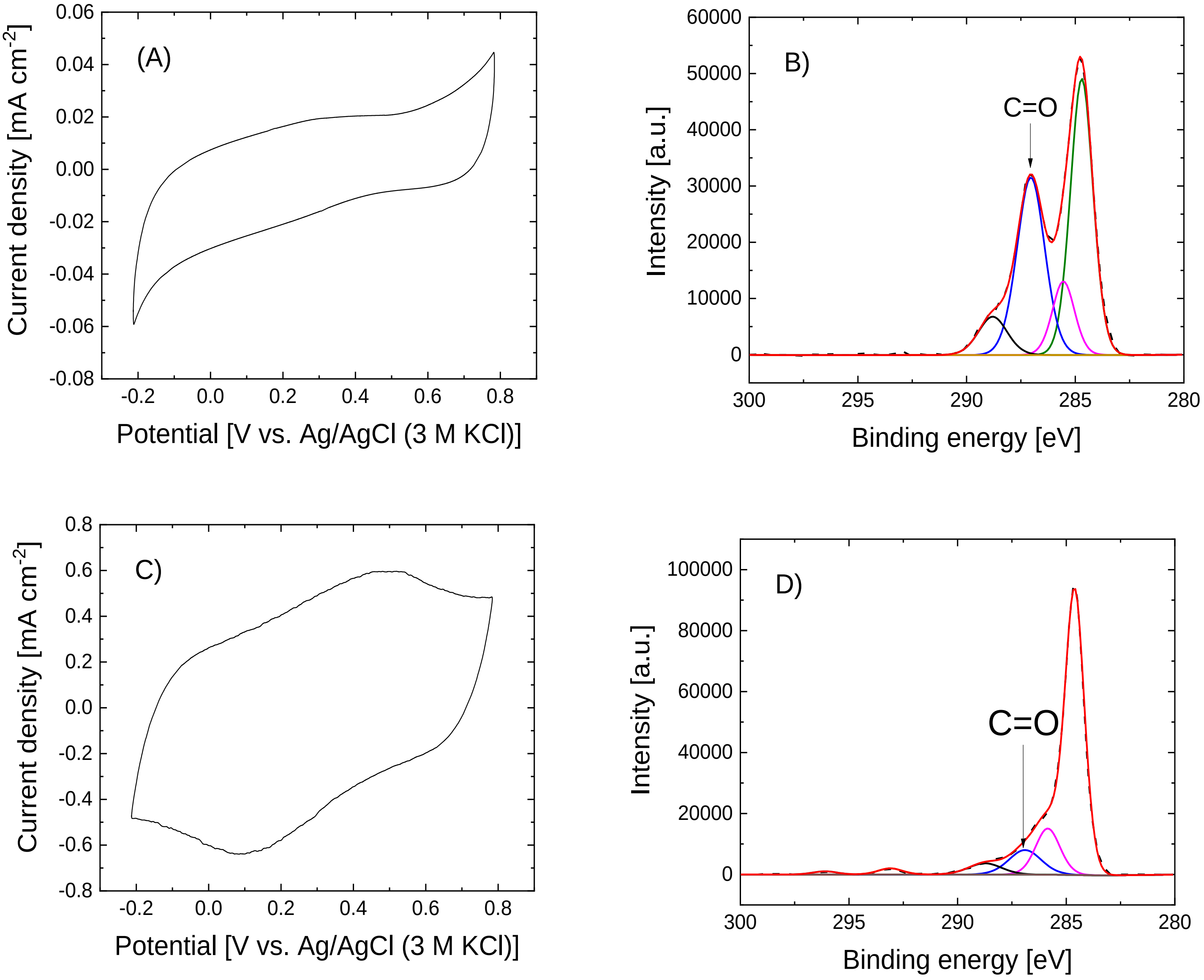

Fig. S1. (A) Cyclic voltammogram for GO in 0.5 M H_2SO_4 at 50 mV s^{-1} under N_2 atmosphere. (B) XPS spectra of C1s signal for GO. (C) Cyclic voltammogram for rGO in 0.5 M H_2SO_4 at 50 mV s^{-1} under N_2 atmosphere. (D) XPS spectra of C1s signal for rGO after the electrochemical reduction.

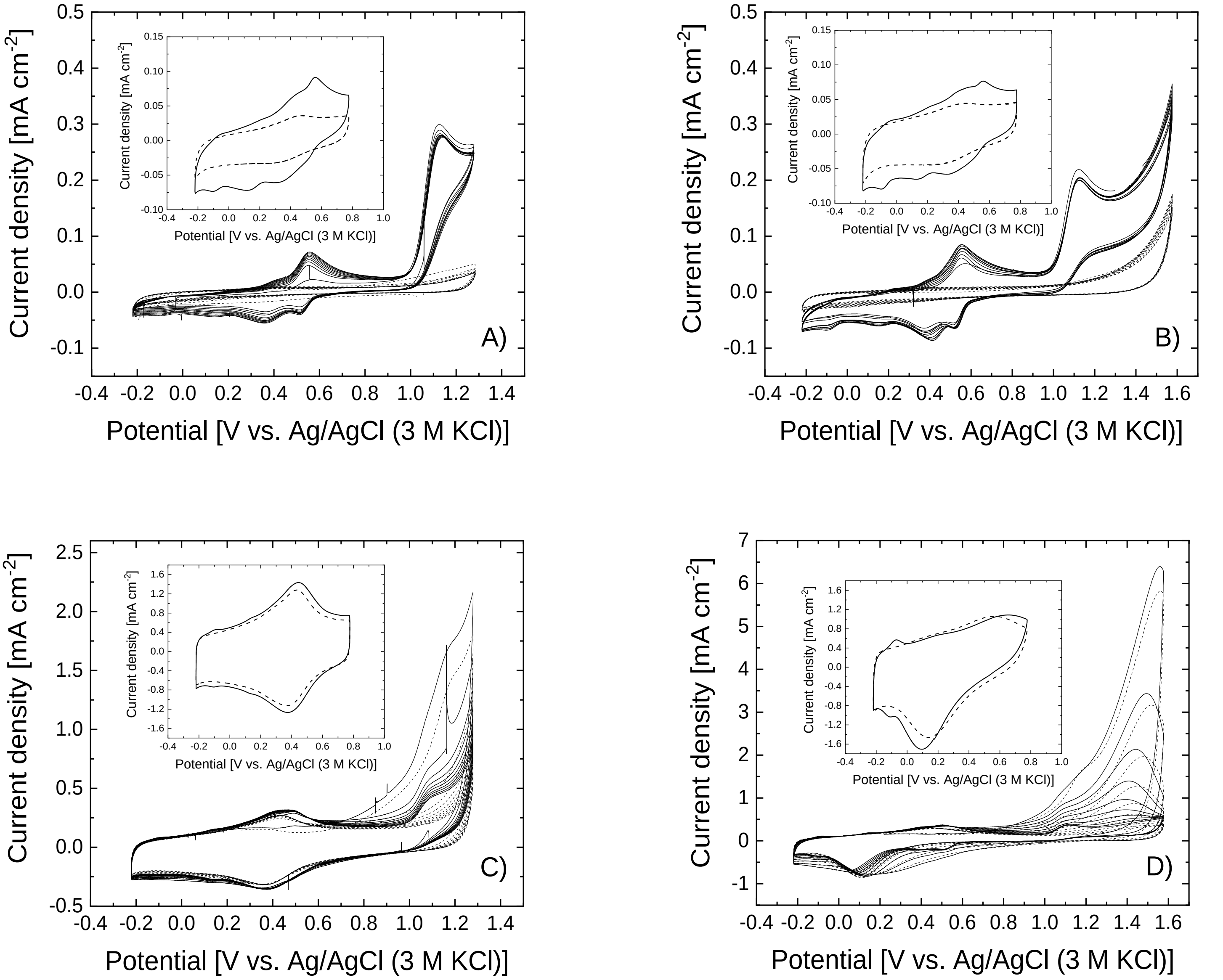

Fig. S2. Cyclic voltammograms obtained during 10 cycles for a GO and rGO electrodes in 0.5 M H_2SO_4 (solid lines) and 0.5 M H_2SO_4 + 1 mM 4-APPA (dash lines) at 10 mV s^{-1} under N_2 atmosphere at different positive potential limits: (A) GO-1.28 V, (B) GO-1.58 V, (C) rGO-1.28 V, and (D) rGO-1.58 V. Inset: Steady state CVs in 0.5 M H_2SO_4, for graphene-based materials electrochemically modified in absence (dash line) and in presence (solid line) at v_{scan}= 50 mV s^{-1}.

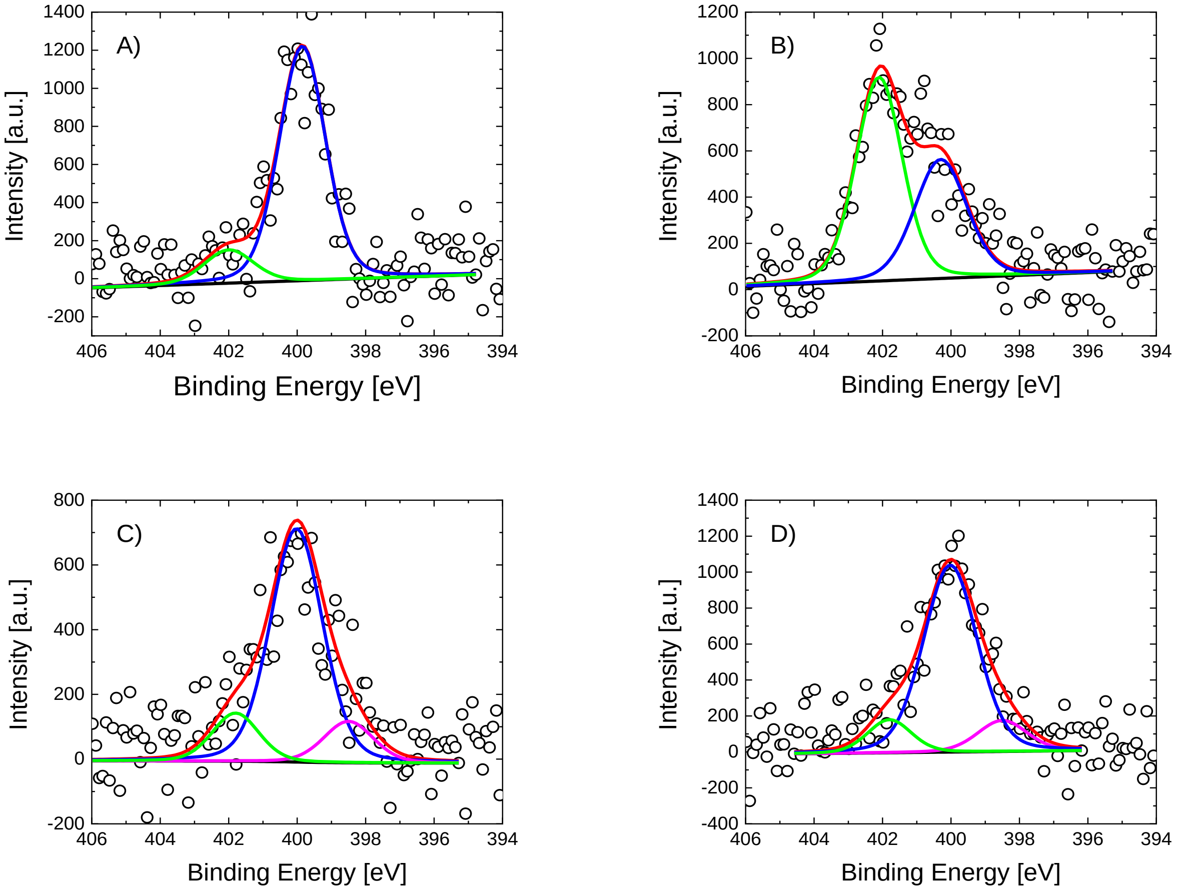

Fig. S3. N1s XPS spectra deconvoluted for GO and rGO electrochemical modified with 4-APPA at different oxidation potential: (A) GO modified at 1.28 V, (B) GO modified at 1.58 V, (C) rGO modified at 1.28 V, and (D) rGO modified at 1.58 V.

Table S1. Chemical distribution of the N and P species obtained from XPS of electrochemical modified graphene-based materials with 4-APPA.

**Graphene-based material**	**Upper potential limit [V vs. Ag/AgCl (3 M KCl)]**	**More oxidized P (134.1 eV)**	**P-O (133.3 eV)**	**C-P (132.6 eV)**	**Imines (398.5 eV)**	**Neutral amines (399.8 eV)**	**Oxidized nitrogen species (~402 eV)**
GO	1.28	-	44	56	--	87	13
	1.58	100	-	-	--	40	60
rGO	1.28	-	66	34	12	74	14
	1.58	-	53	47	11	77	12

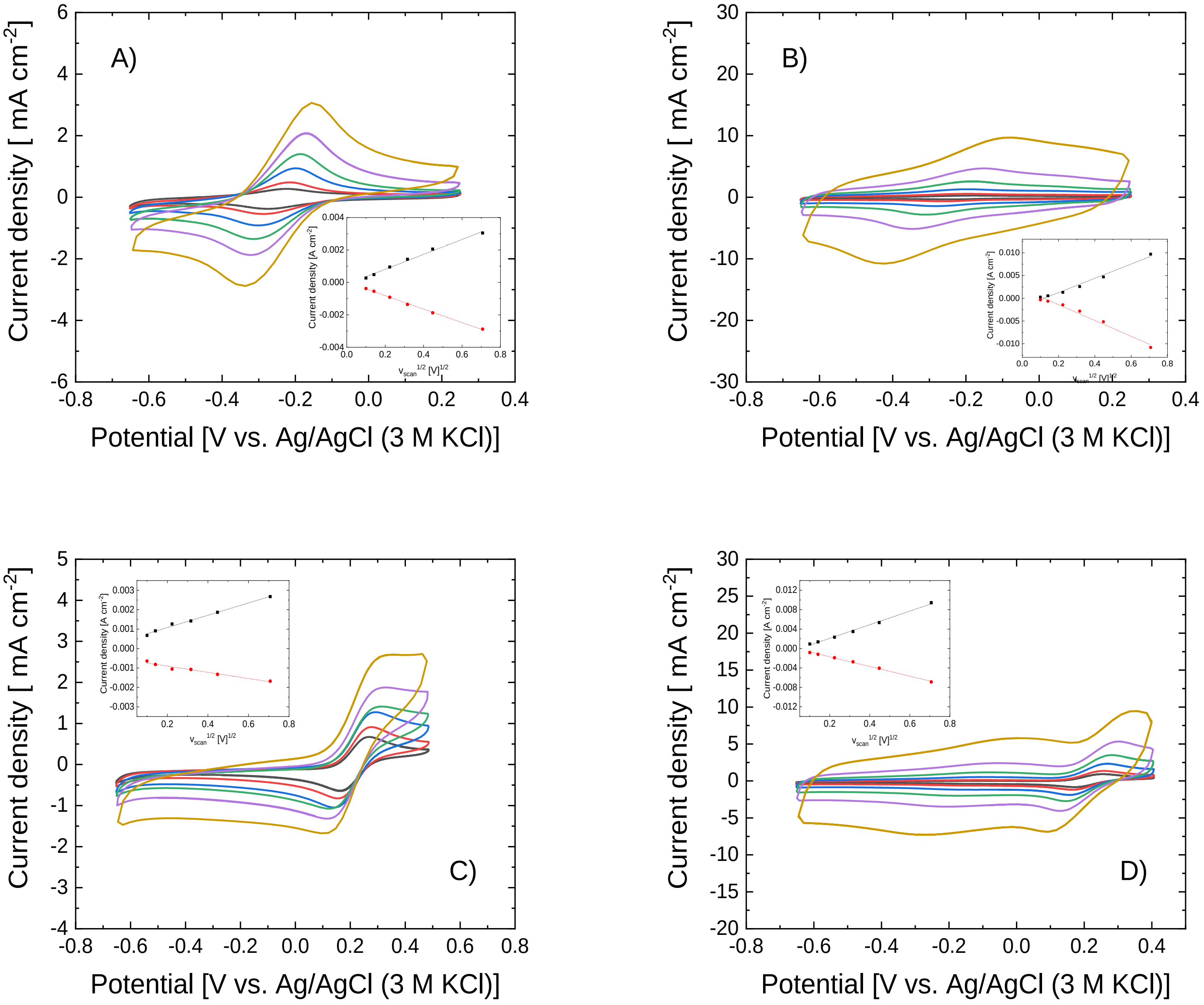

Fig. S4. Cyclic voltammograms at different scan rates (10, 20, 50, 100, 200, and 500 mV s^{-1}) of the pristine graphene-based materials in 0.1 M PBS (pH=7.2): (A-B) 1 mM $[Ru(NH_3)_6]^{3/4+}$ and (C-D) 5 mM ($[Fe(CN)_6]^{3/4-}$).

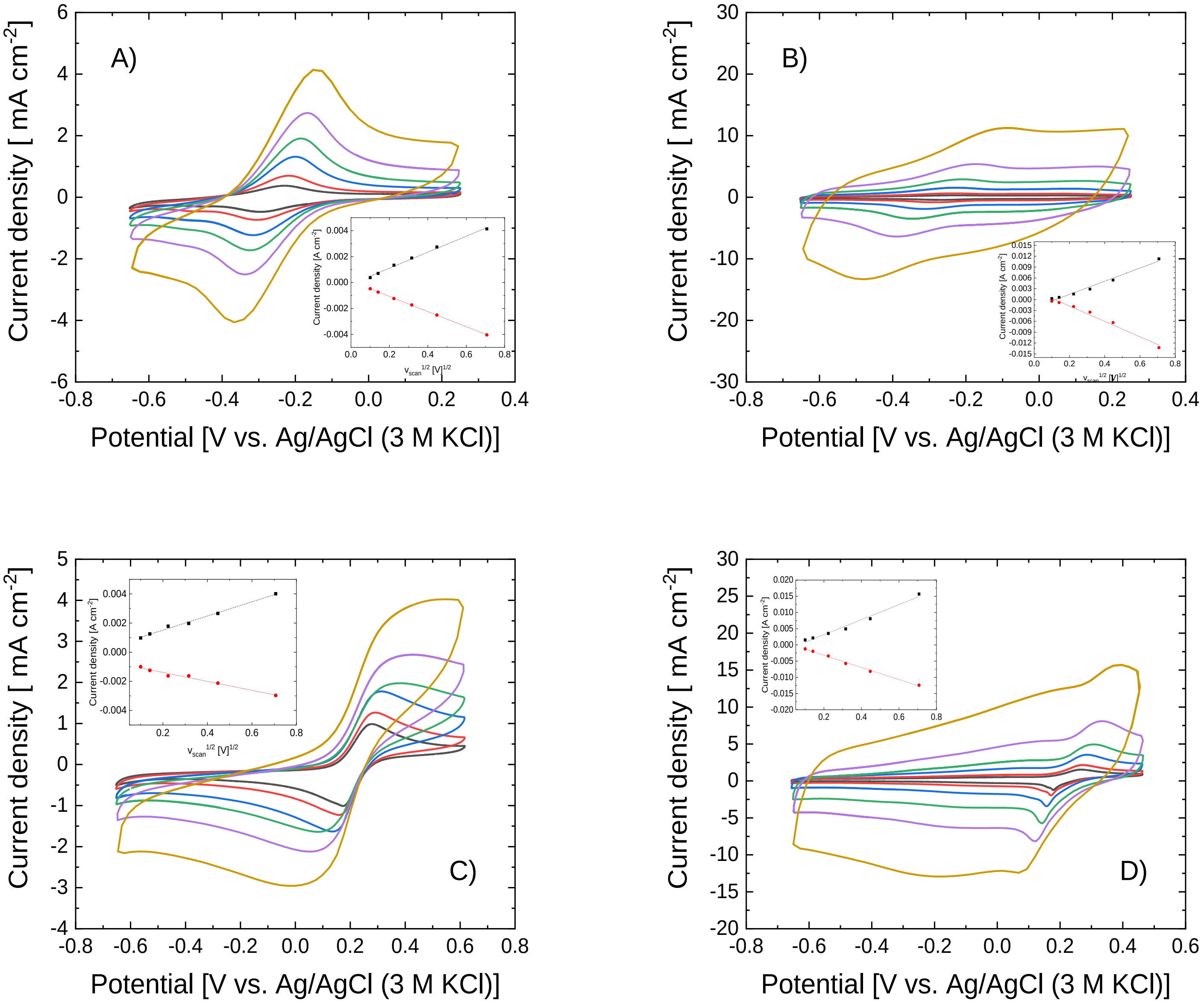

Fig. S5. Cyclic voltammograms at different scan rates (10, 20, 50, 100, 200, and 500 mV s^{-1}) of the graphene-based materials electrochemically modified with 4-APPA in 0.1 M PBS (pH=7.2): (A-B) 1 mM $[Ru(NH_3)_6]^{3/4+}$ and (C-D) 5 mM ($[Fe(CN)_6]^{3/4-}$).

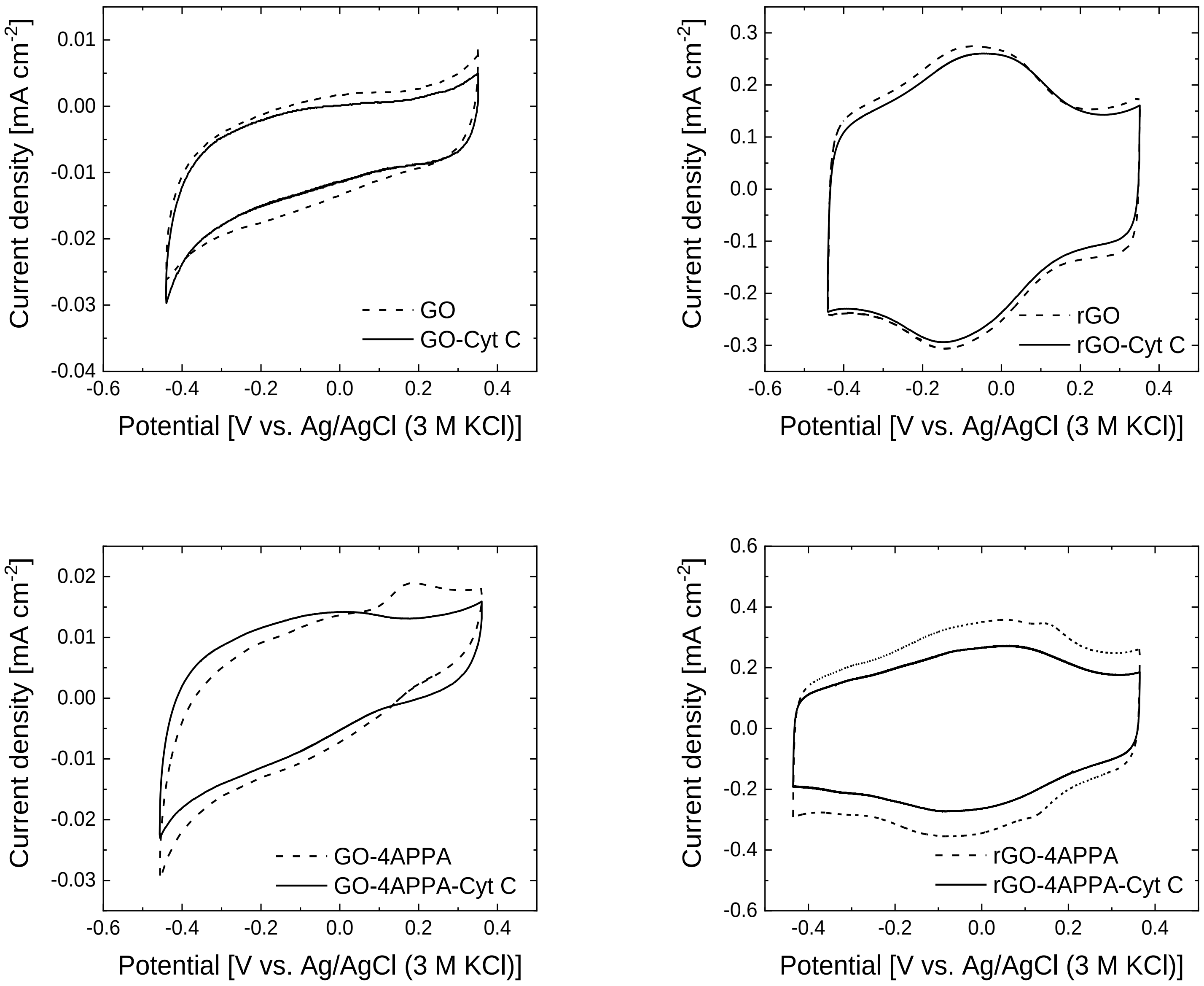

Fig. S6. Cyclic voltammograms of electrochemical modified GO and rGO before and after the immobilization of Cyt C in 0.1 M PBS (pH=7.2) at 20 mV s^{-1}.

Table S2. Comparative of the electrochemical parameters of bioelectrodes reported in literature.

Bioelectrode	LOD [µM]	Sensitivity	Range	Reference
Cyt c/NanoC	--	45 µA/mM	20 to 240 µM	[83]
Cyt c/BDD	0.025	75.6 µA/mM cm^2	1 to 450 µM	[84]
MWCNT@H_2Q-COOH@Cyt c	--	53 µA/mM	0.001 to 1.5 mM	[85]
GCE/CB@NQ/Cyt c	--	0.58 µA/mM	0 to 0.06 mM	[28]
Cyt c/PANI/MWCNTs		32.2 µA/mM	0.5 µM to 1.5 mM	[86]
Si3N4-FET-Pt	10000	--	10 to 100 mM	[87]
Cyt c/MWCNTs	--	83.7 µA/mM	2 to 420 µM	[88]
Cyt c/4-APPA/rGO	100	111.68 µA/mM cm^2	0.1-60 mM	This work

S3. Reference

83. Zhang, L. Direct electrochemistry of cytochrome c at ordered macroporous active carbon electrode. *Biosensors and Bioelectronics* **2008**, *23*, 1610-1615. https://doi.org/10.1016/j.bios.2008.01.022.

84. Zhou, Y.; Zhi, J.; Zou, Y.; Zhang, W.; Lee, S. T. Direct Electrochemistry and Electrocatalytic Activity of Cytochrome c Covalently Immobilized on a Boron-Doped Nanocrystalline Diamond Electrode. *Analytical Chemistry* **2008**, *80*, 4141-4146. https://doi.org/10.1021/ac702417x.

85. Gandhi, M.; Rajagopal, D.; Kumar, A. Molecularly wiring of Cytochrome c with carboxylic acid functionalized hydroquinone on MWCNT surface and its bioelectrocatalytic reduction of H_2O_2 relevance to biomimetic electron-transport and redox signaling. *Electrochimica Acta* **2021**, *368*, 137596. https://doi.org/10.1016/j.electacta.2020.137596.

28. Lavanya, V.; Pavithra, D.; Mohanapriya, A.; Santhakumar, K.; Senthil Kumar, A. A π–π Bonding-Assisted Molecular-Wiring of Folded-Cytochrome c and Naphthoquinone and Its Electron-Relay-Based Bioelectrocatalytic H_2O_2 Reduction Reaction Visualized by Redox-Competitive Scanning Electrochemical Microscopy. *Langmuir* **2023**, *39*, 11556–11570. https://doi.org/10.1021/acs.langmuir.3c00941.

86. Lee, K.; Gopalan, A.; Komathi, S. Direct electrochemistry of cytochrome c and biosensing for hydrogen peroxide on polyaniline grafted multi-walled carbon nanotube electrode. *Sensors and Actuators B: Chemical* **2009**, *141*, 518-525, https://doi.org/10.1016/j.snb.2009.06.039.

87. Seo, H.; Kim, C.; Sohn, B.; Yeow, T.; Son, M.; Haskard, M. ISFET glucose sensor based on a new principle using the electrolysis of hydrogen peroxide. *Sensors and Actuators B: Chemical* **1997**, *40*, 1-5. https://doi.org/10.1016/S0925-4005(97)80191-8.

88. Zhao, G.; Yin, Z.; Zhang, L.; Wei, X. Direct electrochemistry of cytochrome c on a multi-walled carbon nanotubes modified electrode and its electrocatalytic activity for the reduction of H_2O_2. *Electrochemistry Communications* **2005**, *7*, 256-260, https://doi.org/10.1016/j.elecom.2005.01.006.
